# Depuration of *Aliarcobacter butzleri* and *Malaciobacter molluscorum* in Comparison with *Escherichia coli* in Mussels (*Mytilus galloprovincialis*) and Oysters (*Crassostrea gigas*)

**DOI:** 10.3390/pathogens13110973

**Published:** 2024-11-07

**Authors:** Nuria Salas-Massó, Ana Fernández-Bravo, Edgar Bertomeu, Karl B. Andree, Maria José Figueras, Dolors Furones

**Affiliations:** 1 Aquaculture, A Institut de Recerca i Tecnologia Agroalimentàries (IRTA), Centre de la Ràpita, 43540 la Ràpita, Catalonia, Spain; nsalasma@gmail.com (N.S.-M.); edgar.bertomeu@gmail.com (E.B.); karl.andree@irta.es (K.B.A.); 2 Unidad de Microbiología, Departamento de Ciencias Médicas Básicas, IISPV, Facultad de Medicina y Ciencias de la Salud, Universidad Rovira i Virgili, 43201 Reus, Tarragona, Spain; mariajose.figueras@urv.cat

**Keywords:** depuration, *Aliarcobacter*, *M* *alaciobacter*, *Escherichia coli*, mussel, oyster

## Abstract

*Arcobacter*-related species are considered emerging food-borne and waterborne pathogens, with shellfish being a suggested reservoir. In a published study that investigated 204 shellfish samples and 476 isolates, the species *Arcobacter butzleri* (now known as *Aliarcobacter butzleri*) and *Arcobacter molluscorum* (now known as *Malaciobacter molluscorum*) have been isolated as the most dominant species. However, the efficiency of depuration for eliminating *A. butzleri* and *M. molluscorum* in comparison with *Escherichia coli* from mussels and oysters is unknown and is therefore the objective of this investigation. The shellfish depuration process was evaluated in the laboratory, in summer and winter, using mussels and oysters collected from the Ebro Delta harvesting areas after performing a natural contamination and an artificial contamination using the same conditions for both mollusk and seasons. The natural contamination was performed by exposing the shellfish to a freshwater channel that receives untreated sewage from the village of Poble Nou (PNC) and that had a salinity of 10.7–16.8‰. The artificial contamination exposed the shellfish to *A. butzleri* and *E. coli* (in one tank) and to *M. molluscorum* and *E. coli* in another tank under controlled conditions of salinity (34.5‰) and temperature (20 °C summer and 14 °C winter). When evaluating the reduction in the bacteria load (every 24 h) throughout 120 h, the naturally contaminated shellfish at the PNC showed a higher reduction than the shellfish contaminated at the laboratory, with the exception of *M. molluscorum*, that at 24 h could not be detected in summer, neither in mussels nor in oysters. This may be attributed to the fact that the bacteria from the PNC were less adapted to the conditions of high salinity (34.5‰) in which the depuration process was performed. Although temperature did not statistically make a difference in depuration, at 20 °C a higher elimination of all bacteria was recorded relative to 14 °C. In general, *E. coli* survived more in mussels than in oysters, and *M. molluscorum* suffered in both mollusks a higher reduction than *A. butzleri.* New studies are required to determine further the safety of bivalves regarding the presence of *Arcobacter*-related species.

## 1. Introduction

Global aquaculture production reached a record of about 126 million tons in 2021, and in 2023, 18.42 million tons (14.6%) were mollusks [1,2]. According to published FAO and WHO reports [1,2] Spain is the main producer of marine mollusks in Europe, where the most commonly farmed or harvested species are mussels and oysters. The filter-feeding behavior of bivalves makes them vehicles of concentration, accumulation, and dissemination of a diverse number of microorganisms present in the harvesting waters [3,4,5,6,7]. Those microorganisms include pathogenic bacteria, viruses, and protozoa, which either have their origin in terrestrial fecal contamination (i.e., *Salmonella*, *Shigella*, *Arcobacter*, Hepatitis A virus, Norovirus, etc.) or are indigenous from the marine environment, like *Vibrio* species [1,4,6,7,8]. This accumulation together with the fact that bivalve mollusks are usually consumed raw or slightly cooked poses a risk for the consumer [2,5,7].

The sanitary quality of marketable shellfish is routinely monitored by analyzing their levels of fecal contamination using *Escherichia coli* as an indicator of the potential presence of other harmful microorganisms [9,10,11]. In the European Union (EU), it is mandatory to establish a classification of the shellfish harvesting areas based on the levels of *E. coli* found in the shellfish matrix [12,13,14]. Briefly, in class A production areas, a ≤230 Most Probable Number (MPN) of *E. coli*/100 g sets the limit in shellfish to allow them to be placed on the market for consumption. The other classes (B–D) correspond to higher concentrations of *E. coli* in the shellfish; i.e., in class B, 90% of the shellfish samples can hold up to ≤4600 MPN *E. coli*/100 g, and the remaining 10% should not exceed 46,000 MPN/100 g; in class C, all samples can have up to ≤46,000 MPN *E. coli*/100 g and can be placed on the market for human consumption; these shellfish require depuration until they reach the values of category A [12,13,14]. Class D areas include shellfish which are prohibited for consumption because their *E. coli* levels exceed 46,000 MPN/100 g. Shellfish depuration is a process that reduces microbial contaminants (using *E. coli* as an indicator species) by placing shellfish in high-quality seawater for a given time, where they will purge the contaminants until the levels of *E. coli* reach class A status [12,13,14]. The duration of depuration is dependent on the shellfish species, size, and/or their bacterial content. Additionally, environmental factors such as temperature, salinity, and dissolved oxygen are also important for determining the efficacy of the depuration process [15,16,17,18]. It has been demonstrated that *E. coli* may not be a suitable indicator of the presence of pathogens like indigenous *Vibrio*, viruses, or *Arcobacter* spp. [3,4,9,14,17,19,20]. This is mainly caused by the ability of these pathogens to colonize shellfish [9,19] and to survive in shellfish hemocytes [21].

As summarized in the review by Salas-Massó et al. [20], the genus *Arcobacter* has been isolated from shellfish worldwide, mussels being the most studied, followed by clams and oysters. The more frequently recovered species from shellfish have been *Arcobacter butzleri*, which is capable of causing diarrhea and bacteremia in humans, and its presence in shellfish may have a fecal origin [7,14,20,22,23,24,25,26]. After *A. butzleri* (60.2%), the second most prevalent species in a study that investigated 204 shellfish samples and identified 476 isolates was *Arcobacter molluscorum* (21.2%), and both species predominated in the samples recovered from June to October from the Ebro Delta shellfish growing area [22].

In 2018, the genus *Arcobacter* was divided into seven different genera: *Arcobacter*, *Aliarcobacter*, *Pseudarcobacter*, *Halarcobacter*, *Malaciobacter*, *Poseidonibacter,* and Candidate “*Arcomarinus*” gen. nov., according to an exhaustive taxonomic study performed by Pérez-Cataluña et al. [27]. The species *A. butzleri* has become a member of the genus *Aliarcobacter*, comprising other pathogenic species like *Aliarcobacter cryaerophilus* and *Aliarcobacter skirrowii*, and now *A. molluscorum* is a member of the genus *Malaciobacter*, which comprises species mainly recovered from bivalves and marine environments [27,28,29]. The habitats of these new genera, i.e., *Malaciobacter*, *Halarcobacter,* or *Poseidonibacter*, which seem to be associated with shellfish microbiota, are still unclear, but a recent hypothesis indicates that some may be opportunistic pathogens of these animals [30,31,32]. The high incidence of pathogenic species like *A. butzleri* has drawn attention to the study of how this particular species is transmitted and incorporated into shellfish tissues [21,23,26]. Therefore, studies on the shellfish depuration parameters and the survival capacity of *Arcobacter*-related species recovered from shellfish and the marine environment are paramount to generate the needed information to set the criteria to guarantee the safety of shellfish regarding these bacteria.

The Ebro Delta River region is a major producer of mollusks in Spain, cultivating mainly Mediterranean mussels (*Mytilus galloprovincialis)* and Pacific oysters (*Crassostrea gigas*), and it is classified as a B area according to EU regulation 854/2004 [12,13]. In fact, the majority of *Arcobacter* species recovered from shellfish have been described from this area [7,11,14,22]. Considering this together with the lack of studies involving depuration strategies for *Arcobacter*, the objectives of this study are: (i) to analyze how *Arcobacter*-related species, in comparison to *E. coli*, are depurated in mussels and oysters from this harvesting area at two different temperatures (summer and winter) under experimental natural or artificial contamination conditions and (ii) to establish if the potentially pathogenic species *A. butzleri* and the environmental species *M. molluscorum* show any significant differences in their depuration rate [12,13,14].

## 2. Materials and Methods

### 2.1. Sample Collection

A total of 10 kg of mussels (*M. galloprovincialis*) and 40 kg of oysters (*C. gigas*) were bought on arrival at the same local depuration establishment in July and December 2015 to guarantee that the shellfish were collected from the area, represented the production zone microbiological levels and had the market size. The mussels and oysters were analyzed for the quantification of *E. coli* and *Arcobacter*-related spp. by means of MPN, to establish the background of the bacteria load prior to the depuration trial.

### 2.2. Bacterial Culture Preparation

The strains *Aliarcobacter butzleri* (F146-25) and *Malaciobacter molluscorum* (F146-34) were both isolated from the same mussel of the species *M. galloprovincialis* from the Ebro Delta in a previous study [14]. The *E. coli* CECT 434 (WDCM 00013) strain used for the co-contamination experiment is a clinical strain that is used as a quality-reference strain in Food and Water Microbiology (https://www.uv.es/uvweb/coleccion-espanola-cultivos-tipo/es/cect/catalogo-cepas/medios-cultivo/buscador-cepas-1285892802374.html, accessed on 29 September 2024).

Each strain was grown separately on Blood Agar (BA; TSA supplemented with 5% sheep blood BD Difco, Le Pont de Claix, France) at 30 °C for 48 h in aerobic conditions. For *E. coli*, the culture was prepared on Tryptone Soy Agar (TSA; Oxoid, Basingstoke, UK) and was incubated at 37 °C for 24 h. After incubation, a single colony of each *Arcobacter*-related species was individually grown in 100 mL of *Arcobacter* broth supplemented with cefoperazone, amphotericin B, and teicoplanin (Arcobacter-CAT broth; Oxoid, Basingstoke, UK) at 30 °C for 48 h, while *E. coli* was grown in Tryptone Soy Broth (TSB Oxoid Basingstoke, UK) and incubated at the same conditions mentioned above. Pure cultures of the three species were transferred to sterile centrifuge tubes and centrifuged at 3000× *g* for 15 min. Pelleted cells from each culture were washed twice with 50 mL of sterile saline solution (0.9% NaCl *w*/*v*; SSE).

The suspensions used as inoculums for the co-contamination assays were prepared individually for each species, *A. butzleri* and *M. molluscorum*, and contained 0.8 L of SSE inoculated using a bacterial suspension with a final OD at 550 nm of 0.8, which we had predetermined corresponds with a bacterial load of 10^6^ CFU/mL for both *A. butzleri* and *M. molluscorum*. For *E. coli*, a bacterial suspension of 10^5^ CFU/mL was used, obtained by equilibrating to a McFarland standard of 1 (bioMérieux, Marcy l’Etoile, France).

### 2.3. Experimental Design

Two assays were performed, one in summer (July) and one in winter (December), under the same conditions. The shellfish (mussels and oysters), previously cleaned, were placed in different fiberglass tanks (90 × 45 cm), with a total volume of 40 L. The water supply consisted of a flow-through system, fed with seawater from Alfacs Bay, which had been previously sterilized by passage through a device equipped with filtration (5 µm pore diameter) and ultraviolet light.

**Figure 1:**
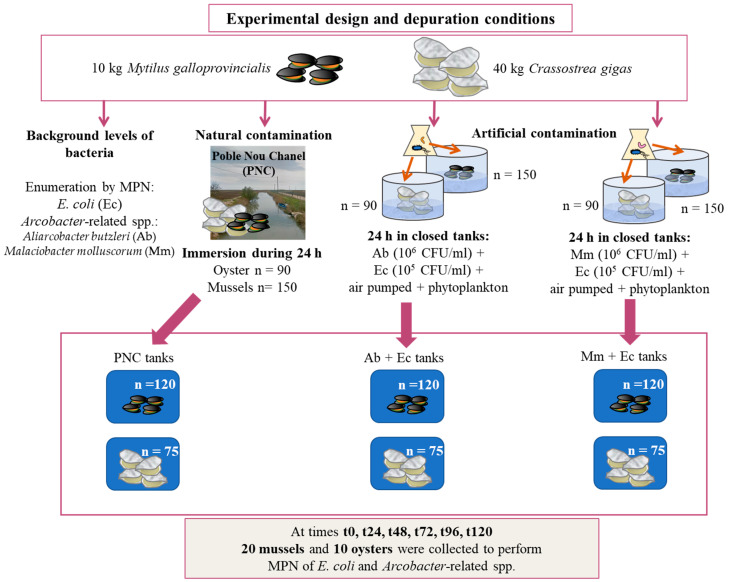
Scheme of the different experimental conditions that were used in this study.

The temperature and salinity of the depuration tanks in the IRTA facilities had an average temperature of 20 °C and 14 °C in summer and winter, respectively, with a salinity for both periods of 34.5‰. The inlet water was microbiologically analyzed to corroborate the efficacy of the described disinfection process. The water renewal rate was 0.7 L/min. To avoid recontamination with the feces of the shellfish, a perforated elevated plastic grill was placed at the bottom of all the tanks. Shellfish were not fed during the depuration process that was carried out for 5 days using 6 different tanks in summer and winter, each of them containing 120 mussels and 75 oysters (Figure 1).

Naturally contaminated Poble Nou Chanel (PNC) tanks: one contained mussels and the other one oysters that were previously naturally contaminated by direct immersion during 24 h in the freshwater channel that receives untreated sewage from the village of Poble Nou (Tarragona, Spain), which in a previous study showed levels of *E. coli* corresponding to a harvesting area of class D (4.1 × 10^4^ ± 3.6) and equivalent high loads of *Arcobacter* spp. of 4.5 × 10^5^ ± 9.3 [14]. The purpose was to have shellfish highly polluted with naturally occurring bacteria. The temperature of the PNC water during the 24 h exposure time was 26.8 °C and 9.7 °C in summer and winter, respectively, and presented a salinity of 10.7‰ and 16.8‰, respectively. After the natural bioaccumulation process, the shellfish were washed with tap water to remove mud on the shells and were placed in their depuration tanks.

Artificial contamination tanks: one contained mussels and the other one oysters that were contaminated by direct immersion in one tank with cultures of both *E. coli* (Ec) and *A. butzleri* (Ab) and another with cultures of *E. coli* (Ec) and *M. molluscorum* (Mm), as shown in Figure 1. They were maintained in closed contamination tanks for 24 h while air was pumped to keep the dissolved oxygen level favorable for the animals, which were fed with axenic phytoplankton (10^5^ cells/mL of *Isochrysis galbana*) to stimulate the uptake of the bacteria [33]. The axenic conditions of the phytoplankton, concerning *E. coli* and *Arcobacter*-related spp., were verified with MPN cultures, the results of which were negative for both microbes. After the artificial contamination, the animals were washed and transferred to the depuration tanks, where they were kept unfed for 5 days under the same conditions described above, namely: a water renewal rate of 0.7 L/min, at 20 °C and 14 °C in summer and winter, respectively, and a constant salinity for both periods of 34.5‰. Additional oxygen was not needed for the biomass of the tanks.

### 2.4. Microbiological Analysis

After the 24 h exposure time under each condition, a quantification of the initial bacterial load (CFU/100 g and MPN/100 g), time 0 (t0), was performed for each group of contaminated shellfish, and thereafter the shellfish were placed in the depuration tanks and microbiological analyses were performed at times 24 h, 48 h, 96 h, and 120 h. Briefly, flesh and intervalval liquid from 20 mussels and from 10 oysters were placed in separated stomacher bags with peptone water and were mixed thoroughly and homogenized in a stomacher (Lab·Blender 400, West Sussex, UK). Then, 100 g of the homogenate was used for preparing the three dilutions (i.e., 1, 0.1, and 0.01 mL or g of the original sample) that were used in the two-step MPN for *E. coli* and *Arcobacter*-related spp. For *E. coli*, the quantification was performed as described previously [14]. Briefly, five tubes, in triplicates containing Glutamate broth (Oxoid, Basingstoke, UK), were inoculated with the dilutions of the homogenates and incubated for 24 h at 37 °C. Confirmation of *E. coli* was performed from the tubes that changed color from purple to yellow, by subculturing a loop of cells from the Glutamate broth tubes onto Tryptone Bile X-glucuronide Agar (TBX) medium (Oxoid, Basingstoke, UK) at 44 °C for 24 h. The growth of typical greenish-blue-color colonies confirmed the presence of *E. coli*. The derived MPN results were calculated using the Centre for Environment Fisheries and Aquaculture Science (CEFAS) MPN tables (https://www.cefas.co.uk/bacteriological-contamination-of-bivalves/methods/, accessed on 10 February 2024).

For *Arcobacter* quantification, a two-step MPN was performed, according to Salas-Massó et al. [14]. Briefly, five tubes, in triplicates, containing *Arcobacter*-CAT enrichment broth, were inoculated with previously prepared serial dilutions and incubated at 30 °C for 48 h, under aerobic conditions. Confirmation of the presence of *Arcobacter*-related spp. was performed by inoculating 0.1 mL of the above series of enrichment tubes, after passive filtration (0.45 µm nitrocellulose filters; Millipore, Darmstadt, Germany) on Blood Agar (BA) plates at 30 °C for 48 h under aerobic conditions, and the observation of the typical small, beige to off-white, translucent and convex colonies as well as the typical small curved-rod morphology of cells seen after Gram staining. The MPN final values were obtained using the software MPN Build 23 (Mike Curiale software; http://i2workout.com/mcuriale/mpn/index.html, accessed on 20 April 2024).

To corroborate that the quantification of *A. butzleri* and *M. molluscorum* corresponded to the strains used in the bioaccumulation process, eight colonies of the previously mentioned cells were resuspended in 0.1 mL aliquots subcultured on BA and were then selected for genotyping. Thereafter, DNA was extracted from each of the colonies to perform the Enterobacterial Repetitive Intergenic Consensus PCR (ERIC-PCR) genotyping technique [22]. The ERIC-PCR patterns were then compared with those obtained from strains F146-25 and F146-34 used in the initial inoculum

### 2.5. Statistical Analysis

The results of the MPN were transformed into log values, and statistical analyses were performed with Software SPSS Statistical 22.0 (IBM Analytics). Normality distribution of the data was assessed using the Shapiro–Wilk and Kolmogorv–Smirnov test. Bacterial populations in oysters and mussels at different sampling times and at different temperatures were analyzed with the non-parametric Mann–Whitney test for non-normal distributed data, and significant differences between the compared data means of treatments were established at a *p* value ≤ 0.05.

## 3. Results

**Figure 2:**
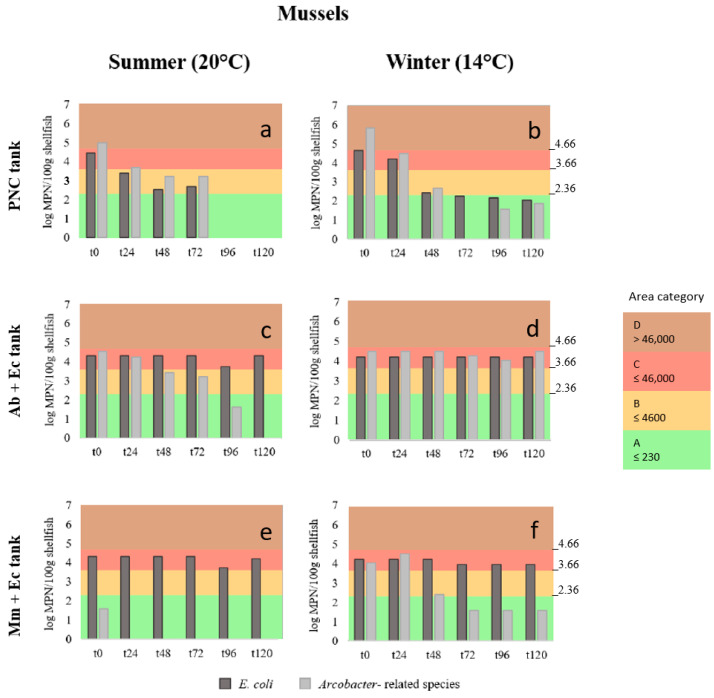
(**a**–**f**) Bar representation of the log MPN/100 g of *Escherichia coli* (Ec) and *Arcobacter*-related species (Ab, *Aliarcobacter butzleri* and Mm, *Malaciobacter molluscorum*) found in the naturally contaminated (at the Poble Nou channel, PNC) and the artificially contaminated mussels, in summer and winter, in the different trial depuration tanks (**a**–**f**) and obtained from time 0 (t0) every 24 h up to 120 h. The different colors indicate the standards of the four categories (A, B, C and D) established by the European Union for shellfish harvesting areas based on the MPN results of *E. coli*/100 g [12,13]: class A (green), ≤230 MPN/100 g (log 2.36)—shellfish do not require depuration; class B (orange), ≤4600 MPN/100 g (log 3.66); class C (red), ≤46,000 MPN/100 g (log 4.66) and class D (brown), ≥46,000 MPN/100 g—these shellfish are prohibited for consumption. For human consumption Class B to C require depuration to reach class A.

**Figure 3:**
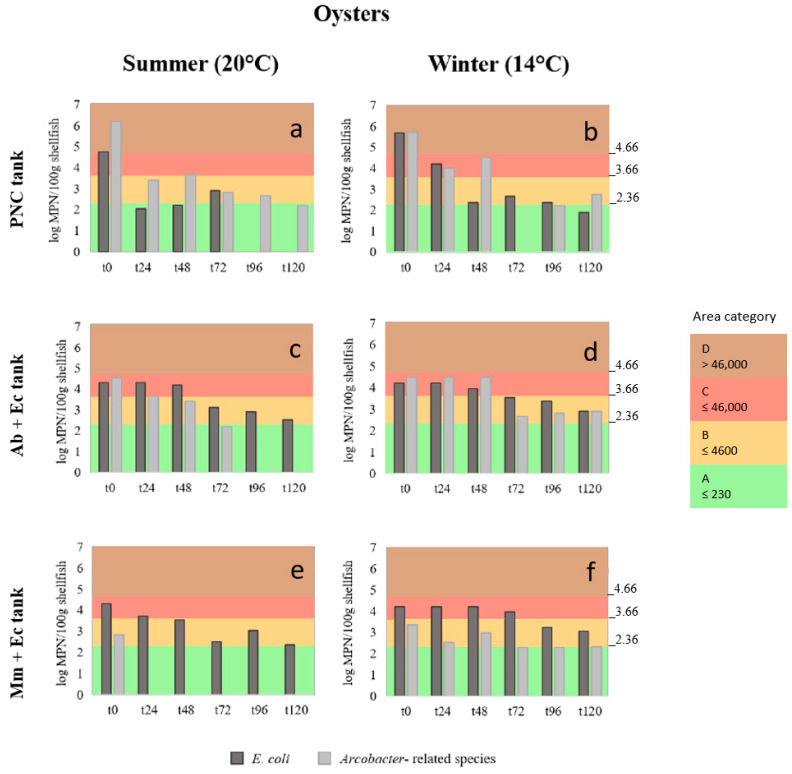
(**a**–**f**) Bar representation of the log MPN/100 g of *Escherichia coli* (Ec) and *Arcobacter*-related species (Ab, *Aliarcobacter butzleri* and Mm, *Malaciobacter molluscorum*) found in the naturally contaminated (at the Poble Nou channel, PNC) and the artificially contaminated oysters, in summer and winter, in the different trial depuration tanks (**a**–**f**) and obtained from time 0 (t0) every 24 h up to 120 h. The different colors indicate the standards of the four categories (A, B, C and D) established by the European Union for shellfish harvesting areas based on the MPN results of *E. coli*/100 g [12,13]: class A (green), ≤230 MPN/100 g (log 2.36)—shellfish do not require depuration; class B (orange), ≤4600 MPN/100 g (log 3.66); class C (red), ≤46,000 MPN/100 g (log 4.66) and class D (brown), ≥46,000 MPN/100 g—these shellfish are prohibited for consumption. For human consumption Class B to C require depuration to reach class A.

The shellfish batches locally produced showed a background load of *E. coli* in mussels of 2200 MPN/100 g in summer and 1700 MPN/100 g in winter, while in oysters, taken from a different site, the levels of *E. coli* were <18 MPN/100 g (below the detection limit of the MPN) in summer and 20 MPN/100 g in winter (). This background load of *E. coli* in mussels and oysters corresponds to the levels of class B of the Ebro Delta shellfish growing area, but after 24 h of natural and artificial contamination (t0) reached levels of *E. coli* of ca. >4600 MPN/100 g and ≤46,000 MPN/100 g in summer and winter, respectively, which correspond to the class C growing area, as shown in the Supplementary Tables S1 and S2 and Figure 2a–f and Figure 3a–f. The only exception was the oysters that in summer and winter, after the natural contamination at the PNC, showed levels that corresponded to category D (>46,000 MPN/100 g). All the remaining *E. coli* levels registered at t0 in mussel and oysters fell into C class, regardless of the group treatment or temperature (Figure 2c–f and Figure 3c–f and Supplementary Tables S1 and S2).

In the tanks that contained shellfish naturally contaminated in the PNC, the levels of *E. coli* decreased progressively over time, reaching in summer and winter levels close to or equal to class A (≤230 MPN/100 g), as shown in Figure 2a and Figure 3a,b. Moreover, a total removal of this bacteria in mussels and oysters was observed at t96 in summer (temperature of the water was 20 °C). Total elimination also occurred for *Arcobacter*-related spp. in mussels (Figure 2a), whereas in oysters, the latter bacteria persisted until the end of the trial (Figure 3a). During the winter experiment, *E. coli* decreased in mussels 2.61 log and in oysters 3.80 log, while *Arcobacter*-related spp. showed a decrease in both mussels and oysters, of 3.96 log and 2.97 log, respectively (Figure 2b and Figure 3b and Supplementary Tables S1 and S2). In both shellfish in winter, *Arcobacter*-related spp. were below the detection limit at t72, despite being detected the two following days (Figure 2b and Figure 3b).

The bacterial loads of *E. coli* in mussels, in combination with the *Arcobacter*-related species after the artificial contamination at the laboratory, did not show a significant decrease (Figure 2c–f). However, depuration of *A. butzleri* at 20 °C in mussels occurred progressively from t48 to t96, showing no detection of this bacteria at t120 (Figure 2c). The same happened in the case of the oysters in summer, but the progressive reduction occurred already at t24 with total disappearance at t96 (Figure 3c). In fact, after 48 h, both bacteria started decreasing their load up to 1.30 and 1.78 log in the case of *E. coli* at 14 °C and 20 °C, respectively, and 1.60 log for *A. butzleri* at 14 °C (Figure 3d).

Concerning the mussels and oysters contaminated with *M. molluscorum* and *E. coli,* the dynamics of reduction in the former species was very drastic during the summer experiment at 20 °C (Figure 2e,f). In this case, the initial loads (t0) of *M. molluscorum* in both shellfish were lower than expected, and after 24 h this bacterial species was (Figure 2 and Figure 3) not detected from the animals (Figure 2e and Figure 3e). However, at 14 °C (winter) the levels of *M. molluscorum* in mussels were higher at t0_,_ and in addition, increased slightly (0.46 log) during the first 24 h and decreased strongly during the following 48 h, achieving a final reduction of 2.44 log which was maintained up to t120 (Figure 2f). In the case of oysters, the reduction in *M. molluscorum* in winter was lower (1.02 log), and after 72 h the bacterial concentration was stable during the rest of the trial (Figure 3f). However, *E. coli* barely decreased in mussels either in summer or winter (Figure 2e,f). In the case of the oysters, the *E. coli* reduction achieved loads that corresponded to class B areas, and the MPN values at 20 °C, at the end of the experiment (t120), were very close (280 MPN/100 g) to the limit accepted for class A (≤230 MPN/100 g; Figure 3e).

## 4. Discussion

The abundance of *Arcobacter*-related species in commercialized shellfish, together with the fact that *A. butzleri* is considered an emerging food-borne pathogen and that, to our knowledge, only one study has investigated the bioaccumulation and depuration of the latter species in mussels [1,2,14,21,34,35,36,37], were the reasons that motivated the present study. Our study investigated the experimental depuration of *E. coli*, *A. butzleri,* and *M. molluscorum* in mussels and oysters collected from the Ebro River Delta shellfish production area. Statutory mollusk depuration is the most common procedure worldwide for the purging of bacteria from commercial bivalves before placing them on the market for human consumption [16,17,18,19,38]. However, it has been reported that shellfish depuration is not always 100% efficient at eliminating bacteria, including pathogenic microorganisms; i.e., *E. coli* and *Vibrio* spp. may persist in bivalves after depuration [16,19]. Furthermore, in a study that investigated the bacteria present in the gills of two types of oyster, *C. gigas* and *C. virginica,* harvested from commercial farms in Canada and that related their presence with spoilage, it was found that among the potential pathogenic bacteria, the *Arcobacter*-related species were dominating, and this occurred in oysters harvested at different sites [39]. In conclusion, these microbes were considered the most important gill-spoilage bacteria during refrigerated storage. Thus, this study was undertaken to understand the growth and survivorship of this group of bacteria under experimental depuration conditions.

The performance of the experimental depuration of *E. coli* and *Arcobacter*-related spp. in both shellfish naturally contaminated by immersion in the PNC water was superior, in summer and winter, to the one observed from the artificially contaminated bivalves using bacteria strains under controlled conditions. Thus, higher logarithmic reductions were achieved in the PNC tanks than in the experimentally contaminated shellfish with pure culture mixtures of *E. coli* and one of the two *Arcobacter*-related species tested, which could be attributed to the different origins of the strains involved in the trials. The predominant species of *Arcobacter* identified in the same PNC in a previous study were closely related to those found in association with fecal pollution, like *A. butzleri*, *A. cloacae*, *A. defluvii*, *A. ellisii* and *A. cryaerophilus* [8,9,14]. Therefore, it could be assumed that when the shellfish were exposed to the heavily contaminated freshwater channel (PNC) that showed *E. coli* levels typical of class C and even D, the *Arcobacter* species presented in those samples could be the same as those in the previous studies mentioned above [14]. However, when the shellfish exposed to the PNC were introduced in the depuration system, and therefore in a more saline environment, the persistence and survival of the *Arcobacter* spp. could have been affected, favoring its depuration, because their physiological status might have been compromised. The relationship between the environmental conditions, especially salinity, has been pointed out in our previous studies [10,11,14]. As we indicated, in the controlled experimental exposures, the *A. butzleri* and *M. molluscorum* strains used for contaminating the shellfish were originally isolated from resident bivalves harvested in the same area (Alfacs Bay), and it is, therefore, highly probable that these strains may have mechanisms that help them to persist in high-salinity environments, like shellfish tissues. Interestingly, in this work, we observed that, after 120 h in clean water, the *E. coli* loads from samples from the PNC either were under the detection limit or reached the level of class A. However, the loads in the tanks in which the shellfish were artificially contaminated with *E. coli* with initial levels typical for class C or B areas did not show a significant reduction. Again, this may be due to an effect of the strain used, because culture collection strains may accumulate mutations making these isolates behave differently from the environmental strains [40]; for this reason, different *E. coli* strains should be screened for their depuration performance to properly select those more suitable for these types of experiments. In fact, in the only study of bioaccumulation performed by Ottaviani et al. [26], using the type strain of *A. butzleri,* the authors demonstrated that this strain did not survive in seawater for more than 48 h, and within the contaminated mussels, the concentration decreased ca. 2 log 24 h after the contamination and thereafter 1 log every 24 h up to 96 h. Interestingly, in our depuration study using a mussel-associated strain of *A. butzleri* (from the same area), the strain showed a very low reduction after 24 h and 1 log reduction at 72 h, but total disappearance did not occur up to 120 h (Supplementary Table S1). In our study, the levels of *E. coli* from PNC shellfish samples decreased over the 120 h of the depuration assay at 14 °C (winter temperature) until they reached levels that complied with the legal limits for consumption. However, in summer, the PNC shellfish samples did not hold detectable levels of *E. coli* after 72 h for either mussels or oysters. The same behavior was observed for the *Arcobacter*-related species, which disappeared in the mussels and remained at lower concentrations in the oysters. Such kinetic depuration (total elimination after 72 h) was not observed in any of the shellfish (mussels and oysters) experimentally contaminated by immersion in bacteria cultured at the laboratory.

In our study, the depuration experiments were performed during two different seasons, summer and winter, to understand whether the temperature could influence the depuration rates of the bacteria. Although the statistical test did not prove any significant difference between depuration at 14 °C or 20 °C, it was observed that at 20 °C a higher number of tanks achieved a total reduction in bacterial loads as compared to depuration at 14 °C. In fact, in a previous study, it was demonstrated that levels of *E. coli* in mussels and oysters correctly predicted the presence of the potentially pathogenic species *A. butzleri* and *A. cryaerophilus* when the shellfish were harvested from water with temperatures lower than 26.2 °C, thus failing to act as a good indicator above these temperatures [14]. Other authors have reported that temperature influences the depuration rates. Shen et al. [19] found that depuration of *V. parahaemolyticus* in oysters was enhanced at temperatures between 5 °C and 15 °C, rather than at ambient temperatures like 22 °C. Instead, for F+ coliphages, depuration was more effective at 18 °C than at 9 °C [15], and in fact, higher temperatures also favored the depuration of noroviruses [41]. Therefore, to achieve a consensus on the optimal temperature to be set in the depuration process, the growth characteristics of the main pathogens that have to be eliminated should be taken into consideration. More comparisons such as these herein need to be documented. However, physicochemical parameters (i.e., temperature and salinity) are important to ensure the veracity and quality of the experiments, because any stress to shellfish should be avoided when establishing these parameters to maintain conditions as close as possible to their values in their harvesting areas [18]. As this may occur in other studies, it should be considered that the experimental results obtained in our study could represent an overestimation or underestimation of the depuration efficiency for these bacteria in the real world, i.e., the industrial commercialized depuration, considering the high diversity of naturally occurring strains of those species in shellfish. The potential differences, as discussed above, can be associated with the strains used in the experiments and with the experimental scale of the depuration system vs. the industrial one. The possibility of a potential receptor interference between the *E. coli* and *Arcobacter*-related strains used during the depuration process has not been explored in the present study, and this possibility should be taken into consideration in future studies.

To date, there have been few reports of foodborne illness caused by *Arcobacter*, usually associated with consumption of fecally contaminated water or chicken meat, mainly involving *A. butzleri* or *A. cryaerophilus* [20,23,25,42]. However, the first bacteremia causing febrile symptoms, attributed to *Malacobacter mytili* and considered to be produced by direct contact with seafood (Maryland crabs), affected a 65-year old American male, who already had several underlying illnesses [43]. The route of entry of the bacteria was suspected to be through skin wounds in his hands, from where it probably spread to the blood [43]. Interestingly, this species was first isolated in 2006 [7,44] from commercial mussels harvested in the same study area, the Ebro River delta, where the present study is also being conducted. At that time, it was considered that this species was a natural inhabitant of mussels and seawater, but after the bacteremia case described, we now know that the species is also found in crabs from another continent and can act as an opportunistic pathogenic species. The high prevalence of *Arcobacter*-related species in commercial seafood worldwide [5,20,23,25,26,45] is linked to the high abundance of these bacteria in wastewater and sewage samples collected from very diverse geographical regions [9,46,47,48,49]. It has been indicated that the environmental conditions in the sewer infrastructures allow *Arcobacter* population to grow and thrive in this niche, showing a seasonal variation related to the water temperatures [46]. In fact, in a recent study using DNA metabarcoding analysis, 55–73% of the reads from raw wastewater collected in the spring corresponded to *Arcobacter* spp., the most abundant taxa, while after the wastewater treatment, a clear reduction in the reads was observed (<6% total reads), while in the studied mussels collected during the same period, the reads were <2% [50]. These facts should drive attention to design better strategies for purification, as well as identify better bacterial indicators that ensure the safety of seafood products for the consumer. Future studies should test what should be the best depuration system for ensuring the removal of *Arcobacter*-related species from shellfish while keeping in consideration feasible technical and economic aspects.

This study provides preliminary information to help improve depuration standards and protocols for commercial mussels and oysters, focusing on *Arcobacter*-related bacteria as emergent pathogens for this sector. We based our work-frame on the EU standards which are set for *E. coli* as an indicator of fecal pollution.

## 5. Conclusions

This is the first study investigating the efficiency of depuration of the *Arcobacter*-related species in comparison with *E. coli* in shellfish, and the findings can contribute to providing data on the behavior observed for those bacteria under experimental conditions. Our results showed that the experimental contamination model is critical in this study, where the naturally contaminated shellfish underwent a dynamic depuration process, in contrast with the experimentally contaminated animals, where the depuration was stagnant. Additionally, the selection of the indicator strain for depuration experiments is also critical.

## Data Availability

The data presented in this study are available on request from the corresponding authors.

## Supplementary Materials

The following supporting information can be downloaded at: https://www.mdpi.com/article/10.3390/pathogens13110973/s1, Table S1: Values of the log MPN of *Escherichia coli* (Ec) and *Arcobacter*-related species (Ab, *Aliarcobacter butzleri* and Mn, *Malaciobacter molluscorum*) show in Figure 2a–f found in the naturally contaminated (at the Poble Nou channel, PNC) and the artificial contaminated mussels obtained from 100 g, at time 0 (t0) and every 24 h up to 120 h during the depuration trials performed in summer and winter; Table S2: Values of log MPN of *Escherichia coli* (Ec) and *Arcobacter*-related species (Ab, *Aliarcobacter butzleri* and Mn, *Malaciobacter molluscorum*) show in Figure 2a–f found in the naturally contaminated (at the Poble Nou channel, PNC) and the artificial contaminated oysters obtained from 100 g at time 0 (t0), every 24 h up to 120 h during the depuration trials performed in summer and winter.
