# Supplementary material for: Depuration of Aliarcobacter butzleri and Malaciobacter molluscorum in Comparison with Escherichia coli in Mussels (Mytilus galloprovincialis) and Oysters (Crassostrea gigas)
Source: Pathogens. 2024 Nov 7;13(11):973. doi: 10.3390/pathogens13110973 (PMC11597588; doi:10.3390/pathogens13110973)
Supplement: Supplementary file 1 [file pathogens-13-00973-s001.zip › pathogens-3112180-supplementary.pdf]

Table S1. Values of the log MPN of *Escherichia coli* (Ec) and *Arcobacter*-related species (Ab, *Aliarcobacter butzleri* and Mn, *Malaciobacter molluscorum*) show in Figure 2 (a-f) found in the naturally contaminated (at the Poble Nou channel, PNC) and the artificial contaminated mussels obtained from100 g, at time 0 (t0) and every 24 h up to 120 h during the depuration trials performed in summer and winter.

Mussels (Figure 2 a-f)						
Summer (20ºC)						
	PNC tank (Fig. 2a)		Ab + Ec tank (Fig. 2c)		Mm + Ec tank (Fig. 2e)	
	log MPN Ec	log MPN Arco	log MPN Ec	log MPN Ab	log MPN Ec	log MPN Mm
**t0**	4.45	4.99	4.30	4.54	4.30	1.60
**t24**	3.38	3.68	4.30	4.26	4.30	0.00
**t48**	2.52	3.20	4.30	3.41	4.30	0.00
**t72**	2.69	3.20	4.30	3.20	4.30	0.00
**t96**	-	-	3.73	1.60	3.73	0.00
**t120**	-	-	4.30	0.00	4.20	0.00
Winter (14ºC)						
	PNC tank (Fig. 2b)		Ab + Ec tank (Fig. 2d)		Mm + Ec tank (Fig. 2f)	
	log MPN Ec	log MPN Arco	log MPN Ec	log MPN Ab	log MPN Ec	log MPN Mm
**t0**	4.65	5.82	4.20	4.51	4.20	4.04
**t24**	4.20	4.51	4.20	4.51	4.20	4.51
**t48**	2.43	2.65	4.20	4.51	4.20	2.41
**t72**	2.23	0.00	4.20	4.26	3.96	1.60
**t96**	2.15	1.56	4.20	4.04	3.96	1.60
**t120**	2.04	1.86	4.20	4.51	3.96	1.60

Table S2. Values of log MPN of *Escherichia coli* (Ec) and *Arcobacter*-related species (Ab, *Aliarcobacter butzleri* and Mn, *Malaciobacter molluscorum*) show in Figure 2 (a-f) found in the naturally contaminated (at the Poble Nou channel, PNC) and the artificial contaminated oysters obtained from 100 g at time 0 (t0), every 24 h up to 120 h during the depuration trials performed in summer and winter.

Oysters (Figure 3 a-f)						
Summer (20ºC)						
	PNC tank (Fig. 3a)		Ab + Ec tank (Fig. 3c)		Mm + Ec tank (Fig. 3e)	
	log MPN Ec	log MPN Arco	log MPN Ec	log MPN Ab	log MPN Ec	log MPN Mm
**t0**	4.73	6.20	4.30	4.54	4.30	2.82
**t24**	2.04	3.41	4.30	3.64	3.73	0.00
**t48**	2.23	3.68	4.20	3.41	3.54	0.00
**t72**	2.90	2.82	3.11	2.20	2.52	0.00
**t96**	0.00	2.66	2.90	0.00	3.04	0.00
**t120**	0.00	2.20	2.52	0.00	2.36	0.00
Winter (14ºC)						
	PNC tank (Fig. 3b)		Ab + Ec tank (Fig. 3d)		Mm + Ec tank (Fig. 3f)	
	log MPN Ec	log MPN Arco	log MPN Ec	log MPN Ab	log MPN Ec	log MPN Mm
**t0**	5.69	5.72	4.20	4.51	4.20	3.34
**t24**	4.20	4.04	4.20	4.51	4.20	2.52
**t48**	2.36	4.51	3.96	4.51	4.20	2.96
**t72**	2.66	0.00	3.54	2.66	3.96	2.28
**t96**	2.36	2.20	3.38	2.81	3.23	2.28
**t120**	1.89	2.76	2.90	2.90	3.04	2.32
